# Geometric Morphometric Analysis of Sexual Dimorphism in the Bill of the White Stork (*Ciconia ciconia*)

**DOI:** 10.3390/ani15091312

**Published:** 2025-05-01

**Authors:** Ebuderda Günay, Tomasz Szara, Buket Çakar, Emine İrem Deveci, Ali Serhan Coşkun, Gökhan Gün, Funda Yiğit, Ozan Gündemir, Sokol Duro, Mihaela Claudia Spataru

**Affiliations:** 1 Department of Wild Animal Disease and Ecology, Faculty of Veterinary Medicine, Istanbul University-Cerrahpaşa, 34320 Istanbul, Türkiye; 2 Department of Morphological Sciences, Institute of Veterinary Medicine, Warsaw University of Life Sciences-SGGW, 02-776 Warsaw, Poland; 3 Institute of Graduate Studies, Istanbul University-Cerrahpaşa, 34320 Istanbul, Türkiye; 4 Department of Molecular Biology and Genetics, Bogazici University, 34342 Istanbul, Türkiye; 5 Department of Histology and Embryology, Faculty of Veterinary Medicine, Istanbul University-Cerrahpaşa, 34320 Istanbul, Türkiye; 6 Department of Anatomy, Faculty of Veterinary Medicine, Istanbul University-Cerrahpaşa, 34320 Istanbul, Türkiye; 7 Department of Anatomy, Faculty of Veterinary Medicine, Agricultural University of Tirana, 1000 Tirana, Albania; 8 Department of Public Health, Faculty of Veterinary Medicine, “Ion Ionescu de la Brad” Iasi University of Life Sciences, 700489 Iasi, Romania

**Keywords:** allometry, sexual dimorphism, bill morphology, principal component analysis, sex determination

## Abstract

Sexual dimorphism in bill morphology influences foraging strategies and bird competitive interactions. This study uses geometric morphometric analyses to examine sex-based differences in the bill shape and size of *Ciconia ciconia*, a large wading bird. Standardized dorsal and lateral photographs of 45 white storks (24 females and 21 males) were analyzed. The mean centroid size (CS) confirmed that males have significantly larger bills than females, yet principal component analysis revealed no reliable shape-based sex differentiation. Allometric analyses indicated that larger individuals exhibit more pronounced shape variations, likely tied to functional adaptations, though these were insufficient for sex determination. While bill size correlates with sex, shape variation does not serve as a reliable criterion for sexing *C. ciconia*. This study contributes to the understanding of sexual dimorphism in *Ciconia ciconia* by quantifying bill size differences using geometric morphometrics.

## Simple Summary

The white stork (*Ciconia ciconia*), a sexually monomorphic migratory species, uses Türkiye as a key migration route. We employed geometric morphometric methods, analyzing photographs of 45 white storks (24 females and 21 males), to explore sexual dimorphism in bill morphology. Although no significant shape differences emerged between sexes, males exhibited larger bills, and larger individuals displayed more significant shape variation, suggesting an allometric effect. These findings highlight the utility of geometric morphometrics in detecting size-based sexual dimorphism in monomorphic birds.

## 1. Introduction

The white stork (*Ciconia ciconia*), a large migratory bird of the *Ciconiidae* family, inhabits diverse ecosystems across Europe, western Asia, and parts of Africa, with Türkiye serving as a vital migration corridor [1,2,3,4,5]. This species avoids long sea crossings by utilizing the Bosphorus, a natural passage between Europe and Asia, and forages on prey such as frogs, earthworms, fish, and insects during its summer visits and migratory stopovers in Türkiye [4,5]. Understanding the ecological roles of white storks, including their foraging behavior and habitat use, is critical for conservation and requires accurate sex determination to assess sex-specific environmental interactions, such as differential resource use or migratory strategies.

Sexual dimorphism, the morphological difference between males and females, is a key area of research in birds, as it often influences foraging strategies, competitive interactions, and reproductive success [6]. In species like hummingbirds and ducks, dimorphism is evident in plumage and coloration [7,8,9]. Still, in monomorphic species with minimal external differences, morphometric analysis of structures like the beak becomes critical for sex determination and ecological research [10,11]. Geometric morphometrics has proven effective in detecting subtle beak shape variations, as demonstrated in studies of raptors and pigeons, where inter- and intraspecific differences were identified [12,13].

Sex identification in monomorphic birds like the white stork poses challenges for breeding, conservation, and research [14,15]. With identical black and white plumage, red legs and bills, and no vocal songs, white storks lack obvious phenotypic distinctions between sexes [16,17,18]. Consequently, understanding subtle morphological differences, such as those in beak shape, is essential for developing non-invasive sex identification methods and advancing ecological studies of this species. Molecular sexing has been employed [19,20], but non-invasive field methods remain underdeveloped. Although male storks are generally larger than females [16,17,21,22], differences in head and bill measurements are inconsistent [23,24,25]. Additionally, mandible clapping—an acoustic behavior influenced by bill size—exhibits sexual dimorphism, suggesting a functional significance [26,27,28]. The beak, a highly adaptable structure, plays a key role in feeding and ecological interactions shaped by natural and sexual selection [29,30,31]. Sex-specific beak morphology may influence foraging strategies and environmental roles, yet its dimorphism and allometric patterns in white storks are underexplored.

Geometric morphometrics (GM) offers a sophisticated methodology for quantitatively assessing shape variation, leveraging homologous anatomical landmarks to preserve spatial interrelationships. This approach employs Procrustes superimposition to dissociate shape data from confounding variables such as size, position, and orientation. [32,33,34,35,36]. Using principal component analysis (PCA), GM captures and quantifies subtle yet biologically meaningful variations in morphology. [10,37]. Previous studies on other avian species, such as hummingbirds, raptors, and penguins, have successfully used geometric morphometrics (GM) to detect subtle bill shape variations associated with sexual dimorphism [7,12,29,37,38,39]. These methods have also proven effective in examining interspecific variation in closely related species [40]. This study focuses on the bill of the white stork to address the gap in understanding its sexual dimorphism, motivated by the need for non-invasive sex identification methods in field research and conservation. We chose the bill due to its ecological and behavioral significance and measurable morphological variation, which GM can quantify highly [10,35]. Our hypotheses are as follows: (1) male white storks exhibit larger bill sizes than females, which is consistent with general body size dimorphism; (2) bill shape differs between sexes, reflecting functional adaptations such as foraging efficiency or acoustic signaling; and (3) allometric effects influence bill shape, with larger individuals showing more pronounced shape variations. By applying GM to analyze dorsal and lateral bill photographs, this study aims to quantify size and shape differences, assess their reliability for sex determination, and contribute to the development of non-invasive techniques for white stork research.

## 2. Materials and Methods

### 2.1. Animals

Forty-five white stork (*Ciconia ciconia*) skulls were obtained from the Department of Wildlife Diseases and Ecology, Faculty of Veterinary Medicine, Istanbul University-Cerrahpasa. The sample included 24 females and 21 males. During the spring migration season, white storks (*Ciconia ciconia*) that were found exhausted, injured, or weakened were brought to the Wildlife Rehabilitation Center of the Department of Wildlife Diseases and Ecology, Faculty of Veterinary Medicine, Istanbul University-Cerrahpaşa, located in Avcılar, Istanbul. This area lies on one of the main migratory routes for birds. Upon admission, all storks underwent detailed clinical examinations. Individuals with minor injuries or general fatigue were kept under care until their health stabilized. The body weights of all individuals were recorded, with an average weight of 2.9 kg. Once the birds were deemed healthy, they were kept at the center until the next migration season. During this time, they were released by joining naturally passing flocks of storks migrating through the Avcılar region. The birds were housed in species-appropriate outdoor enclosures and received standard supportive feeding and veterinary care. No euthanasia or invasive procedures were performed during the rehabilitation process. Individuals who died due to natural causes or injury-related complications were stored at −20 °C until used for morphometric analyses. All storks underwent clinical examination by specialists, and only adult specimens without pathological lesions were included.

### 2.2. Sex Identification

**Figure 1:**
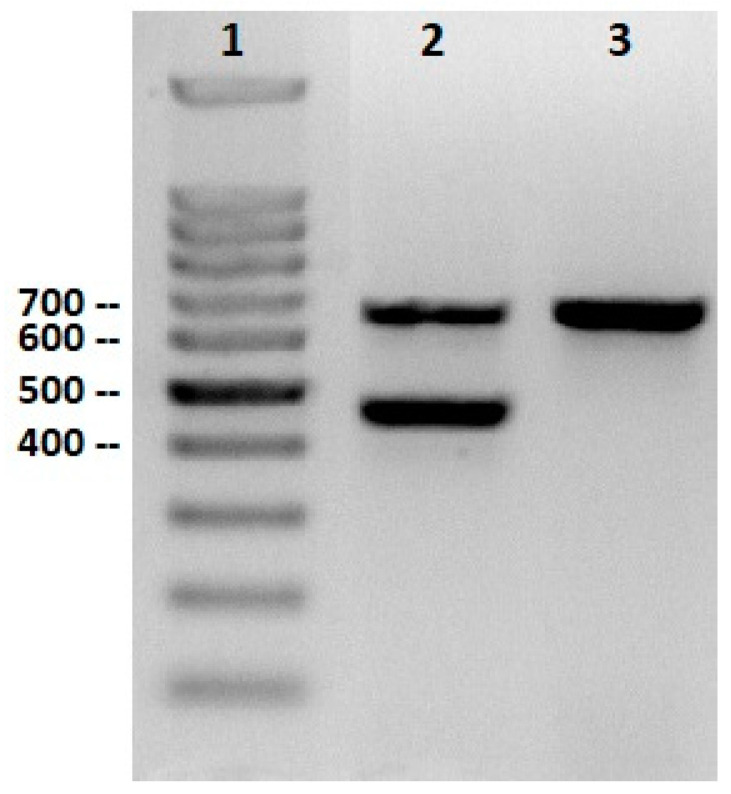
Gel electrophoresis of PCR products using 2550F-2718R primers. Lane 1: 100 bp DNA ladder (Intronbio, Korea); Lane 2: Female (661 bp, 460 bp); Lane 3: Male (661 bp).

The sex of each white stork (*Ciconia ciconia*) was determined using molecular methods described by Fridolfsson and Ellegren [20]. Genomic DNA was extracted from feather calamus samples, and the chromo-helicase-DNA binding (CHD) genes on the Z and W sex chromosomes were amplified via PCR using primers 2550F and 2718R. PCR products were separated by agarose gel electrophoresis, with females showing two bands (661 bp for CHD-W and 460 bp for CHD-Z) and males showing a single band (661 bp for CHD-Z) (Figure 1).

### 2.3. Data Collection and Analysis

**Figure 2:**
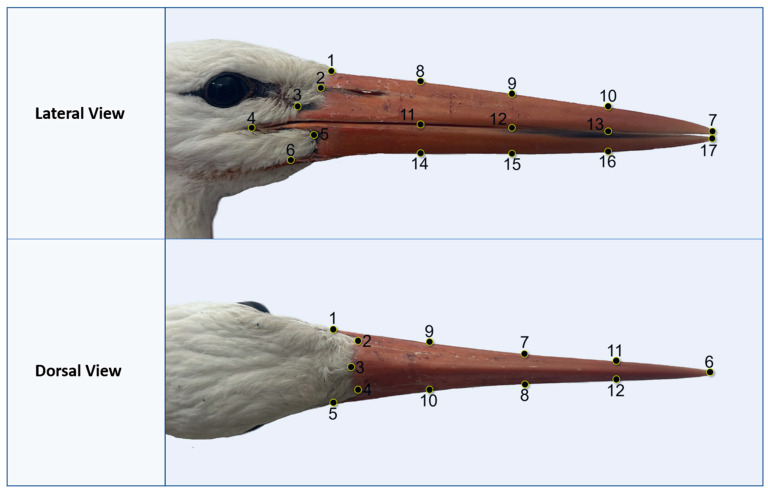
Landmarks for dorsal and lateral views.

Standardized photographs of each skull were taken from dorsal and right lateral views at 20 cm using an iPhone 15 camera. Images were converted to TPS format with tpsUtil (v. 1.82) [41] and digitized with 12 dorsal and 17 lateral landmarks in tpsDig (v. 2.32) [42] (Figure 2).

Geometric morphometric analyses were conducted in MorphoJ (v. 1.08.01) [43]. Generalized Procrustes Analysis (GPA) aligned landmarks, removing scale, rotation, and translation effects [24,44,45,46]. Principal component analysis (PCA) was used to explore and visualize the data’s principal axes of shape variation. Statistical significance of group differences in shape was assessed separately using permutation tests (1000 iterations, *p* < 0.05) on the Procrustes distances. Analysis of Variance (ANOVA) compared centroid size (CS) between sexes, using the sum of squares, F-statistics, and mean squares. Multivariate regression was used to examine allometry, testing the relationship between shape (Procrustes coordinates) and centroid size (CS). Discriminant Function Analysis (DFA) tested shape-based sex differentiation. Analyses were performed in MorphoJ (ver. 2.32) and Past (ver. 4.17), with results visualized via wireframes and tables.

## 3. Results

### 3.1. Size

**Figure 3:**
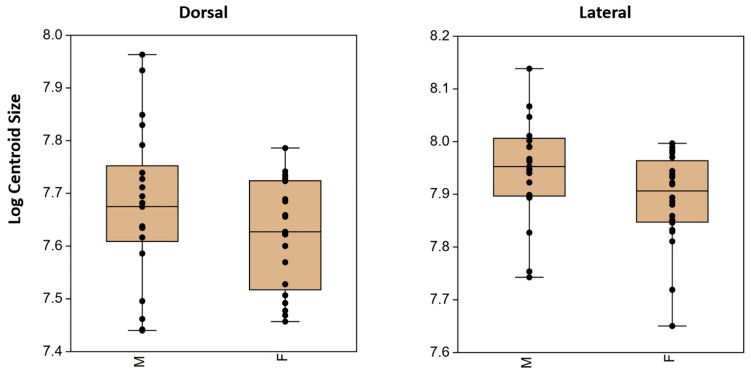
Boxplots of centroid size (CS) for male and female white storks in dorsal (**left**) and lateral (**right**) views, showing larger bill sizes in males, particularly in the lateral view.

Centroid size (CS) analysis, using logarithmically transformed values, was conducted to compare bill sizes from dorsal and lateral views (Figure 3). ANOVA results (Table 1) showed no significant difference in CS between males and females in the dorsal view (*p* = 0.1428, F = 2.2284), but a significant difference in the lateral view (*p* = 0.02935, F = 5.0804), indicating that males have larger bills than females. Boxplots (Figure 3) confirmed this trend, with male CS values consistently higher than female values, particularly in the lateral view. These findings demonstrate size-based sexual dimorphism in bill morphology, especially in the lateral perspective.

### 3.2. Shape

The PCA method was used to understand the shape variation between females and males. Landmark coordinates were used to identify the principal components (PCs) contributing to shape variation. As shown in Table 2, the first three PCs in the dorsal view explained 46.79%, 24.63%, and 11.30% of the variance, respectively, capturing most of the shape variation. In the lateral view, only the first two PCs were considered, accounting for 44.64% and 19.15% of the variance. These two components account for the primary shape variation in the lateral view.

#### 3.2.1. Dorsal View

**Figure 4:**
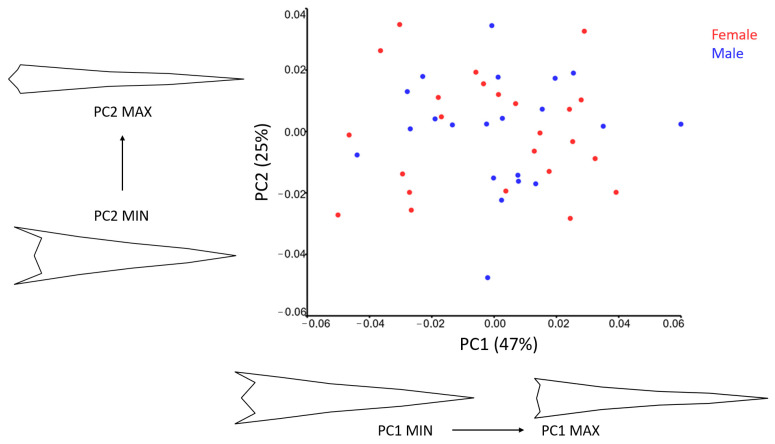
Scatter plot of PC1 and PC2 of the pigeon bill shape in dorsal view for sexes. Wire-frame warp plots of PC1 (47%) and PC2 (25%) of changes in the stork bills, as mapped by 12 LMs, show the shape changes associated with the positive and negative extremes of the PC axes.

The PC1-PC2 scatter plot illustrates the distribution of male and female storks along both axes, showing no clear separation between them, suggesting the absence of a distinct dimorphic pattern in the dorsal view (Figure 4).

Wireframe visualizations reveal that at lower PC1 scores, the lateral parts of the gape are positioned more caudally, the nostrils appear more rostrally within the nasal fossa, and the bill is broader. In contrast, at higher PC1 scores, the bill thins, and the gape’s lateral parts shift rostrally.

For PC2, lower scores correspond to more rostrally positioned nostrils. At higher PC2 scores, the gape is wider, and the overall bill shape is more pronounced in higher PC2 scores.

**Figure 5:**
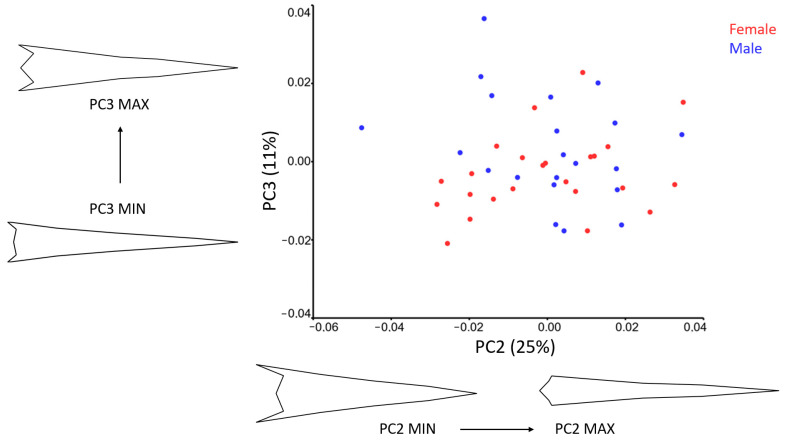
Scatter plot of PC2 and PC3 of the pigeon bill shape in dorsal view for sexes. Wire-frame warp plots of PC2 (25%) and PC2 (11%) of changes in the stork bills, as mapped by 12 LMs, show the shape changes associated with the positive and negative extremes of the PC axes.

The PC2-PC3 scatter plot in Figure 5 shows that males and females are generally mixed with no clear distinction along PC2. However, a recognizable trend can be observed along PC3, where most females are positioned on the negative side of the axis. Males, in contrast, are more dispersed across both the negative and positive sides of PC3.

Wireframe visualizations indicate that PC2 (25%) accounts for a significant portion of shape variation in the dorsal view. Lower PC2 scores are associated with a more caudally positioned gape and a wider bill, whereas higher PC2 scores correspond to a longer and thinner bill with more prominent premaxillary nails.

For PC3 (11%), higher scores are linked to a broader gape and bill with more cranially positioned nostrils, further illustrating shape differences among specimens.

#### 3.2.2. Lateral View

**Figure 6:**
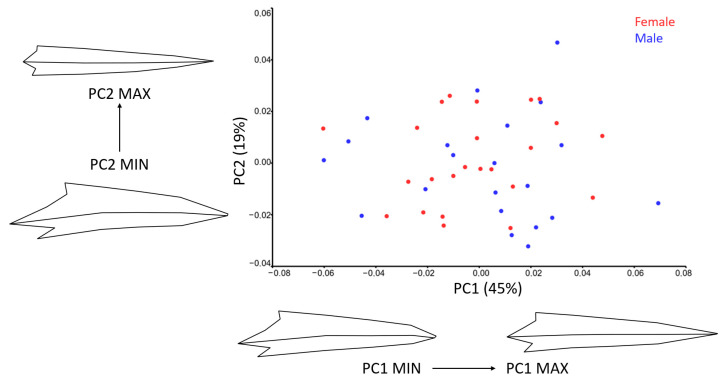
Scatter plot of PC1 and PC2 of the pigeon bill shape in lateral view for sexes. Wire-frame warp plots of PC1 (45%) and PC2 (19%) of changes in the stork bills, as mapped by 17 LMs, show the shape changes associated with the positive and negative extremes of the PC axes.

The PC1-PC2 scatter plot for the lateral view (Figure 6) shows no clear separation between males and females. However, more than half of the males are positioned on the positive side of PC1, suggesting a subtle trend.

Wireframe visualizations indicate that PC1 (45%) captures significant shape variation along the lateral part of the bill. Higher PC1 scores correspond to longer bills with elongated culmens and more pronounced premaxillary and mandibular nails. In contrast, lower PC1 scores are associated with wider bills, a more ventrally positioned premaxillary nail, and a longer, more ventrally located commissure.

PC2 (19%) reflects additional shape differences. Lower PC2 scores correspond to wider bills with a more ventrally positioned base and tip. The premaxillary and mandibular nails are broader, the nares and nasal fossa are more cranio-dorsally positioned, and the commissure is more ventrally positioned. In contrast, higher PC2 scores are linked to straighter culmens.

### 3.3. Discriminant Function Analysis

Discriminant Function Analysis (DFA) showed shape variations between male and female white storks in both dorsal and lateral beak views. The Mahalanobis distance was 0.9809, and the Procrustes distance between the sexes was 0.0077 in the dorsal view, indicating slight variation. Nonetheless, the non-significant *p*-value (0.5843) and the associated T-square value (10.7755) show this difference is not statistically significant. On the other hand, a slightly greater Procrustes distance (0.0113) and a considerably higher Mahalanobis distance (3.6929) were noted in the lateral view, with a T-square value of 152.7424. Despite these more obvious shape differences, the *p*-value (0.1590) stayed above the standard limit point for statistical significance. Based on DFA, findings suggest that, for *Ciconia ciconia*, sex-related shape variation is not significant, even though it is more apparent in the lateral view.

**Figure 7:**
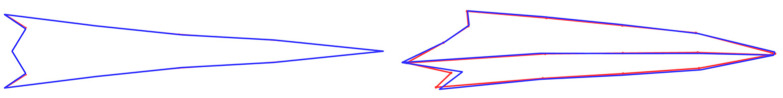
Wire-frame warp plots of shapes of female (red) and male (blue) stork bills from dorsal (**left**) and lateral (**right**) views using discriminant function.

The mean shape wireframes reveal minimal shape differences between male and female storks (Figure 7). In the dorsal view, the Procrustes distance is minimal (0.0077), and the *p*-value is 0.5843, indicating no significant shape differentiation between them (Table 3). The mean shapes of males and females appear nearly identical, with no clear distinction.

In the lateral view, while subtle shape differences exist, such as a slightly more rostral commissure in females and a somewhat more caudal base of the mandible in males, the Procrustes distance remains small (0.0113). The *p*-value (0.1590) further supports the lack of significant shape differences. These findings suggest that their shape differences are minimal and not statistically significant.

### 3.4. Allometry

**Figure 8:**
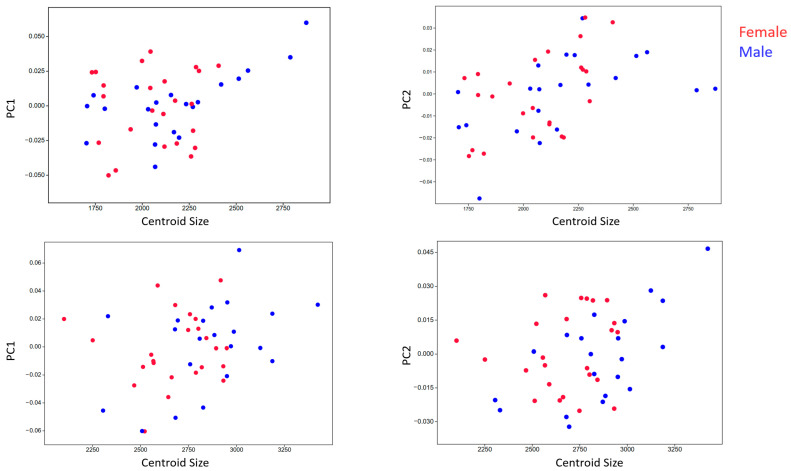
Regression analysis (ordinary least squares regression) on dorsal (top tables) and lateral (bottom tables) shown for both PC1 and PC2 as dependent variables with CS as the independent variable.

Multivariate regression analysis was used to evaluate whether bill shape variation is associated with differences in size (centroid size, CS). This analysis was conducted separately for the dorsal and lateral views (Figure 8).

In the dorsal view, significant relationships were detected between CS and PC1 (*p* = 0.011) and PC2 (*p* = 0.001), indicating that shape variation is partially explained by size, a phenomenon known as allometry. As CS increases, the bill shape changes in the multidimensional shape space; however, this does not imply a linear or directional relationship. Instead, size-related shape change represents a shift in morphological configuration rather than a simple increase or decrease in a specific trait.

In the lateral view, multivariate regression showed a weaker association between CS and PC1 (*p* = 0.056), indicating no statistically significant relationship. However, the analysis revealed a significant association between CS and PC2 (*p* = 0.011), suggesting that shape variation in this view is partially influenced by size. Like the dorsal view, this shape change occurs within a multidimensional morphometric space and should not be interpreted as a linear or directional transformation. The findings support the presence of mild allometric effects in the lateral view.

## 4. Discussion

Sexual size dimorphism (SSD) is common in birds, with males being typically larger than females, giving them an advantage in competing for mates and resources. Darwin suggested that natural selection favored larger and stronger males, enabling them to outcompete rivals during mating and territorial disputes [6].

While SSD has been extensively studied in various bird groups [11,47,48], including large wading birds such as the Saddlebill Stork (*Ephippiorhynchus senegalensis*), where females are noticeably smaller [47], research on other large wading birds, such as herons, ibises, and egrets, has focused on bill-based sexual dimorphism, which has predominantly shown that males tend to have longer bills than females, reflecting sexual dimorphism in bill length [49,50]. Our centroid size (CS) analysis aligns with this trend, showing that males have significantly larger bills than females, particularly in the lateral view, supporting the pattern of sexual size dimorphism (SSD) in storks.

Sexual dimorphism in bill size is crucial in the stork’s foraging behavior and prey handling. Urfi [21] suggested that the larger bill size in male storks may provide an advantage in capturing and handling slippery or resistant prey. A bigger bill allows males to secure a firmer grip, enhancing foraging efficiency, particularly when dealing with prey that could easily slip off. This idea is further supported by Bildstein, who emphasized that larger bills could aid in the more efficient harvesting of food, contributing to males’ fitness [51]. Based on the CS differences observed, allometry appears to play a role in the SSD of white storks. The multivariate regression analyses indicated that some aspects of shape variation are statistically associated with size, particularly in the dorsal view. This suggests that as individuals become larger (typically males), their bill shape changes in specific ways, not linearly or directionally, but within a multidimensional morphospace. These shape changes may reflect functional adaptations related to ecological roles. For instance, Kalam and Urfi [28] reported that Painted Storks forage in shallow water where prey handling time increases with prey size. In such contexts, larger individuals with relatively different bill configurations may be better suited for capturing and processing larger or more mobile prey. In contrast, females with a more petite body and bill sizes may be more efficient at handling smaller prey items. Although our PCA scatter plots show that males and females overlap substantially in shape space, slight tendencies were observed, such as females having lower PC3 scores in the dorsal view, possibly corresponding to differences in bill slenderness. Nonetheless, it is crucial to emphasize that these differences do not imply “larger” or “better” shapes, but somewhat alternative shape configurations likely shaped by ecological and functional pressures.

Differences in bill size between sexes may contribute to functional advantages, such as efficiency in territorial interactions or nest-building activities [52]. These ecological pressures could play a role in shaping size-based dimorphism in storks. While our findings support the existence of size-related differences, it is essential to note that allometric shape changes do not directly indicate advantages or performance traits. Instead, they represent shifts in morphological configuration that may coincide with ecological roles. Assortative mating based on body or bill size may also contribute to these observed patterns, as previously discussed in the literature [49,50,53,54].

Our study identified statistically significant allometric variation in bill morphology, indicating that some aspects of shape change in association with size. Rather than a simple increase or amplification of traits, these changes reflect shifts in morphological configuration within a multidimensional shape space. This observation is consistent with findings by Indykiewicz et al. [55], who described size-associated differences in craniofacial regions that may have ecological relevance. In the lateral view of our PCA plots, males more frequently appeared on the positive side of PC1, which was associated with specific shape features such as an elongated culmen. However, this should not be interpreted as a linear trend or as males “having more” of a trait—instead, these are alternative shape variants. As noted by Bright et al. [56], bill morphology is closely linked to cranial architecture and influenced by allometry, but these shape changes do not follow a simple directional gradient. Our findings agree with this view, illustrating how shape and size covary in complex ways that contribute to—but do not solely define—sexual dimorphism in white storks.

In addition to size-related sexual dimorphism, recent research has highlighted other aspects of sexual dimorphism in storks, particularly acoustic differences and the potential for non-invasive sex identification methods. Eda-Fujiwara [27], along with Urfi and Kalam [28], found that the clattering calls of Oriental White Storks exhibit significant sexual dimorphism in frequency, with males producing lower-frequency calls. This difference is attributed to their larger bill size, which influences sound production mechanics. These acoustic differences could provide a non-invasive approach to sex identification, especially when physical dimorphism is subtle or difficult to observe. Similarly, Cheong et al. [57] suggested that high-quality photographs could be a reliable tool for sexing storks by analyzing morphological indices such as bill size and shape, reporting an 82% accuracy in sex determination using size-related traits. Our findings and analyses further support these results, indicating that while bill size, particularly lateral size, can correlate with sex, morphological shape differences alone do not provide a reliable method for distinguishing in storks. Also, as in a previous study on African Penguins by Szara et al. [39], nearly the same findings as ours were obtained. Males had bigger bills than females and were only significant in the lateral view, just like in our study. These studies also pave the way for future non-invasive, stress-free sex determination in the field, especially with high-quality photographs and advanced geometric morphometric analyses.

Furthermore, despite an 89% success rate in determining the sex of Painted Storks using Discriminant Function Analysis, Cwiertnia et al. [25] found that bill shape and the bill index (BI) were ineffective for sexing or aging White Storks, as bill morphology changes with age. Given the limited knowledge of growth patterns in younger birds, Murata et al. [25] recommended applying this method primarily to storks older than one year. These limitations highlight the need for further investigation into the reliability of these parameters for sex determination.

While this study provides prominent insights into stork bill morphology, it focuses exclusively on shape and size analyses to assess whether these characteristics alone can reliably differentiate between, unlike the previous research by Urfi [21], Cwiertnia et al. [26], and Cheong [57], it is limited by the relatively small sample size and the focus on only two views of the bill. However, nearly the same findings were obtained in a previous study on African Penguins by Szara et al. [38], where males had bigger bills than females and were only significant in the lateral view, just like in our study. They emphasized using geometric morphometric analyses for non-invasive, stress-free sex determination techniques. Using bill morphology alone for sex identification remains challenging, and alternative methods, such as vocalization analysis or high-resolution morphometric assessments, may offer greater accuracy. Future studies should include more individuals, age, and dietary knowledge, and examine additional morphological features to understand bill variation and sexual dimorphism comprehensively.

## 5. Conclusions

This study highlights size-based sexual dimorphism in *Ciconia ciconia* bill morphology, with males exhibiting significantly larger bills than females, as shown by centroid size (CS) analysis, though shape alone is not a reliable method for sex determination. The allometric patterns observed show that larger males exhibit more pronounced shape variations, likely linked to functional adaptations, such as improved prey handling and competitive interactions. However, despite these size differences, our analysis shows that shape variation does not significantly distinguish them. Although male storks have larger bills, differences in bill shapes alone are not reliable indicators of sex.

Therefore, while CS is associated with sex, shape variation does not support sex determination in *Ciconia ciconia*. Future studies that integrate larger datasets, consider age-related variation, examine additional morphological traits across multiple stork species, and use non-invasive methods may improve sex determination techniques and strengthen their applicability in field studies of storks and other large waterbirds.

## Figures and Tables

**Table 1:** ANOVA results comparing centroid size (CS) between males and females in dorsal and lateral views.

	Sum of Squares	Mean Square	F Statistic	*p*-Value
Dorsal	159,819.346	159,819.346	2.2284	0.1428
Lateral	306,785.1804	306,785.1804	5.0804	0.02935

**Table 2:** Result of principal component analysis for dorsal and lateral views.

	Dorsal			Lateral		
*PC*	*Eigenvalue %*	*Variance %*	*Cumulative %*	*Eigenvalues %*	*Variance %*	*Cumulative %*
*PC1*	0.000627	46.788	46.788	0.00080073	44.64	44.64
*PC2*	0.00033007	24.63	71.418	0.0003435	19.15	63.80
*PC3*	0.00015145	11.301	82.719	0.00015325	8.54	72.34

**Table 3:** Procrustes distances and *p*-values among females and males in dorsal and lateral views.

Female-Male	Dorsal	Lateral
Procrustes Distance	0.00765637	0.01131073
*p*-Value	0.5843	0.1590

## Data Availability

The data presented in this study are available upon request from the corresponding authors.

## References

[1] Süel H. (2019). Türkiye’de leylek (*Ciconia ciconia Linnaeus*, 1758) dağılımının iklim değişikliğine göre kestirimi. Turk. J..

[2] Göcek Ç., Çiftçi A., Siki M., Tryjanowski P. (2010). Breeding ecology of the white stork *Ciconia ciconia* in two localities of Turkey. Sandgrouse.

[3] Hall M.R., Gwinner E., Bloesch M. (1987). Annual cycles in moult, body mass, luteinizing hormone, prolactin, and gonadal steroids during the development of sexual maturity in the white stork (*Ciconia ciconia*). J. Zool..

[4] Kahl M.P. (1987). An overview of the storks of the world. Colon. Waterbirds.

[5] Leshem Y., Yom-Tov Y. (1998). Routes of migrating soaring birds. Ibis.

[6] Darwin C. (1874). The Descent of Man and Selection in Relation to Sex.

[7] Berns C.M., Adams D.C. (2012). Becoming different but staying alike: Patterns of sexual size and shape dimorphism in bills of hummingbirds. Evol. Biol..

[8] Sibley C.G. (1957). The evolutionary and taxonomic significance of sexual dimorphism and hybridization in birds. Condor.

[9] Price T.D. (1984). The evolution of sexual size dimorphism in Darwin’s finches. Am. Nat..

[10] Wang C., Fang Z. (2023). Ontogenetic variation and sexual dimorphism of bills among four cephalopod species based on geometric morphometrics. Animals.

[11] Székely T., Lislevand T., Figuerola J., Fairbairn D.J., Blanckenhorn W.U., Székely T. (2007). Sexual size dimorphism in birds. Sex, Size and Gender Roles: Evolutionary Studies of Sexual Size Dimorphism.

[12] Çakar B., Bulut E.Ç., Kahvecioğlu O., Günay E., Ruzhanova-Gospodinova I.S., Szara T. (2024). Bill shape variation in selected species in birds of prey. Anat. Histol. Embryol..

[13] Özkan E., Günay E., Deveci E.İ., Manuta N., Çakar B. (2024). Geometric morphometric analysis of bill shape of Columbimorphae (Columbas, Van, Mardin and Dönek). Anat. Histol. Embryol..

[14] Snow D., Perrins C. (1998). The Birds of the Western Palearctic.

[15] Griffiths R. (2000). Sex identification in birds. Semin. Avian Exot. Pet Med..

[16] Schulz H. (1998). Ciconia ciconia white stork. The Birds of the Western Palearctic: Update 2.

[17] Elliott A., del Hoyo J., Elliott A., Sargatal J. (1992). Family Ciconiidae (storks). Handbook of the Birds of the World, Volume 1: Ostrich to Ducks.

[18] Kazimirski P.P., Kaczmarski M., Zagalska-Neubauer M.M., Żołnierowicz K.M., Tobółka M. (2019). Absence of sex differences in digit ratio in nestlings of the White Stork Ciconia ciconia, a monomorphic bird species. Bird Study.

[19] Purwaningrum M., Nugroho H.A., Asvan M., Karyanti K., Alviyanto B., Kusuma R., Haryanto A. (2019). Molecular techniques for sex identification of captive birds. Vet. World.

[20] Fridolfsson A.K., Ellegren H. (1999). A simple and universal method for molecular sexing of non-ratite birds. J. Avian Biol..

[21] Urfi A.J. (2011). Sexual size dimorphism and mating patterns. The Painted Stork.

[22] Hancock J.A., Kushlan J.A., Kahl M.P. (1992). Storks, Ibises and Spoonbills of the World.

[23] Komiya T., Sugita H., Matsushima K. (1986). Sex determination in the eastern white stork, Ciconia ciconia boyciana, by morphological measurement. J. Jpn. Assoc. Zool. Aquar..

[24] Murata K., Miyashita M., Nagase K., Komiya T., Matsushima K. (1988). Sex determination in the eastern white stork, Ciconia c. boyciana, by bill measurements and discriminant analysis. J. Jpn. Assoc. Zool. Aquar..

[25] Cwiertnia P., Kwieciński Z., Kwiecińska H., Wysocki A., Tryjanowski P., Olsson O. (2006). White Stork Study in Poland: Biology, Ecology and Conservation.

[26] Castiglioni R., Santoro R. Sexual dimorphism in the acoustic signal of the European white stork (*Ciconia ciconia,* L. 1758): A pilot study. Proceedings of the XI Convegno Nazionale Della Ricerca Nei Parchi.

[27] Eda-Fujiwara H., Yamamoto A., Sugita H., Takahashi Y., Kojima Y., Sakashita R., Ogawa H., Miyamoto T., Kimura T. (2004). Sexual dimorphism of acoustic signals in the oriental white stork: Non-invasive identification of sex in birds. Zool. Sci..

[28] Kalam A., Urfi A.J. (2008). Foraging behaviour and prey size of the painted stork. J. Zool..

[29] Foster D.J., Podos J., Hendry A.P. (2008). A geometric morphometric appraisal of bill shape in Darwin’s finches. J. Evol. Biol..

[30] Schoener T.W. (2011). The newest synthesis: Understanding the interplay of evolutionary and ecological dynamics. Science.

[31] Grant P., Grant R. (2008). How and Why Species Multiply: The Radiation of Darwin’s Finches.

[32] Rohlf F.J., Marcus L.F. (1993). A revolution in morphometrics. Trends Ecol. Evol..

[33] Rohlf F.J., Slice D. (1990). Extensions of the Procrustes method for the optimal superimposition of landmarks. Syst. Zool..

[34] Bookstein F.L. (1991). Morphometric Tools for Landmark Data: Geometry and Biology.

[35] Klingenberg C.P. (2010). Evolution and development of shape: Integrating quantitative approaches. Nat. Rev. Genet..

[36] Slice D.E. (2001). Landmark coordinates aligned by Procrustes analysis do not lie in Kendall’s shape space. Syst. Biol..

[37] Fang Z., Chen X., Su H., Thompson K., Chen Y. (2017). Evaluation of stock variation and sexual dimorphism of bill shape of neon flying squid, *Ommastrephes bartramii*, based on geometric morphometrics. Hydrobiologia.

[38] Szara T., Günay E., Boz I., Batmankaya B., Gencer H., Gün G., Vatansever Çelik E.C., Gündemir O. (2023). Bill shape variation in African penguin (*Spheniscus demersus*) held captive in two zoos. Diversity.

[39] Benítez H. (2013). Sexual dimorphism using geometric morphometric approach. Sexual Dimorphism.

[40] Tyler J., Hocking D.P., Younger J.L. (2023). Intrinsic and extrinsic drivers of shape variation in the albatross compound bill. R. Soc. Open Sci..

[41] Rohlf F.J. (2004). TpsDig, version 1.4.

[42] Rohlf F.J. (2006). TpsDig, Digitize Landmarks and Outlines.

[43] Klingenberg C.P. (2011). MorphoJ: An integrated software package for geometric morphometrics. Mol. Ecol. Resour..

[44] Dryden I.L., Mardia K.V. (1998). Statistical Shape Analysis.

[45] Floate K.D., Fox A.S. (2000). Flies under stress: A test of fluctuating asymmetry as a biomonitor of environmental quality. Ecol. Appl..

[46] Klingenberg C.P., McIntyre G.S. (1998). Geometric morphometrics of developmental instability: Analyzing patterns of fluctuating asymmetry with Procrustes methods. Evolution.

[47] Savaş T., Erdem H. (2022). Sexual dimorphism in body size and some exterior traits of pigeon breed groups. J. Poult. Res..

[48] Selander R.K. (1966). Sexual dimorphism and differential niche utilization in birds. Condor.

[49] Sandercock B.K. (1998). Assortative mating and sexual size dimorphism in Western and Semipalmated Sandpipers. Auk.

[50] Chardine J.W., Morris R.D. (1989). Sexual size dimorphism and assortative mating in the Brown Noddy. Condor.

[51] Bildstein K.L. (1987). Energetic consequences of sexual size dimorphism in white ibises (*Eudocimus albus*). Auk.

[52] Vergara P., Gordo O., Aguirre J.I. (2010). Nest size, nest building behaviour and breeding success in a species with nest reuse: The white stork Ciconia ciconia. Ann. Zool. Fenn..

[53] Wagner R.H. (1999). Sexual size dimorphism and assortative mating in razorbills (*Alca torda*). Auk.

[54] Helfenstein F., Danchin E., Wagner R.H. (2004). Assortative mating and sexual size dimorphism in black-legged kittiwakes. Waterbirds.

[55] Indykiewicz P., Manuta N., Bonecka J., Gündemir O., Szara T. (2025). Is the skull of the black-headed gull dimorphic? Analysis of shape variation. Eur. Zool. J..

[56] Bright J.A., Marugán-Lobón J., Cobb S.N., Rayfield E.J. (2016). The shapes of bird bills are highly controlled by nondietary factors. Proc. Natl. Acad. Sci. USA.

[57] Cheong S., Sung H.-C., Park S.-R. (2007). A new method for sexing oriental white storks. J. Field Ornithol..

